# From protein structure to function via single crystal optical spectroscopy

**DOI:** 10.3389/fmolb.2015.00012

**Published:** 2015-04-28

**Authors:** Luca Ronda, Stefano Bruno, Stefano Bettati, Paola Storici, Andrea Mozzarelli

**Affiliations:** ^1^Department of Neurosciences, University of ParmaParma, Italy; ^2^Department of Pharmacy, University of ParmaParma, Italy; ^3^National Institute of Biostructures and BiosystemsRome, Italy; ^4^Elettra Sincrotrone TriesteTrieste, Italy; ^5^Institute of Biophysics, Consiglio Nazionale delle RicerchePisa, Italy

**Keywords:** protein crystal, microspectrophotometry, conformational changes, X-ray crystallography, metastable intermediate, structure-function relationship, synchrotron source

## Abstract

The more than 100,000 protein structures determined by X-ray crystallography provide a wealth of information for the characterization of biological processes at the molecular level. However, several crystallographic “artifacts,” including conformational selection, crystallization conditions and radiation damages, may affect the quality and the interpretation of the electron density maps, thus limiting the relevance of structure determinations. Moreover, for most of these structures, no functional data have been obtained in the crystalline state, thus posing serious questions on their validity in infereing protein mechanisms. In order to solve these issues, spectroscopic methods have been applied for the determination of equilibrium and kinetic properties of proteins in the crystalline state. These methods are UV-vis spectrophotometry, spectrofluorimetry, IR, EPR, Raman, and resonance Raman spectroscopy. Some of these approaches have been implemented with on-line instruments at X-ray synchrotron beamlines. Here, we provide an overview of investigations predominantly carried out in our laboratory by single crystal polarized absorption UV-vis microspectrophotometry, the most applied technique for the functional characterization of proteins in the crystalline state. Studies on hemoglobins, pyridoxal 5′-phosphate dependent enzymes and green fluorescent protein in the crystalline state have addressed key biological issues, leading to either straightforward structure-function correlations or limitations to structure-based mechanisms.

## Introduction

**Figure 1:**
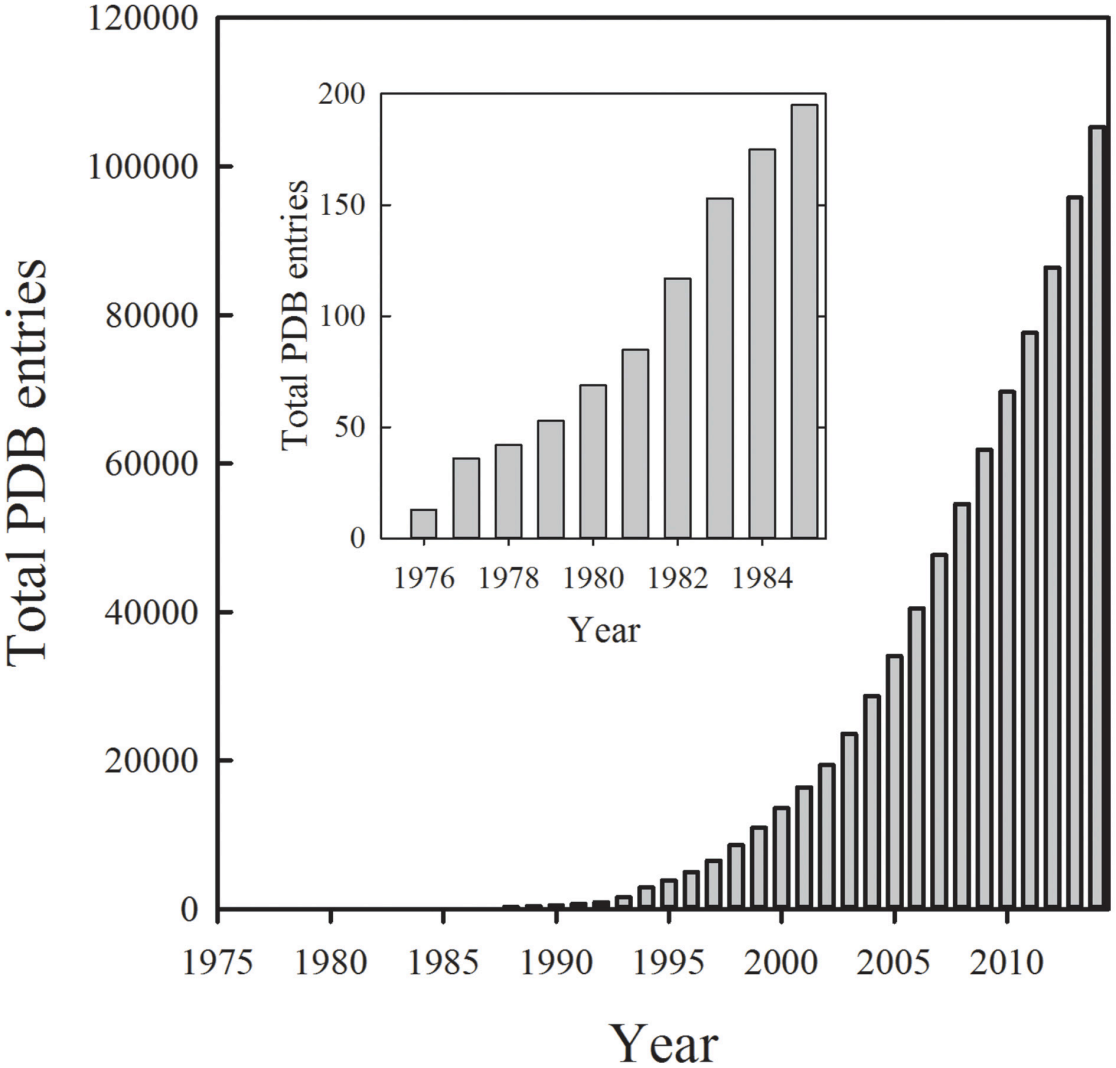
**Cumulative number of protein structures deposited in the Protein Data Bank per year up to the end of 2014 (www.pdb.org)**.

Structural biology is significantly contributing to the current goal of unveiling the molecular bases of biological processes ranging from cell life to cell death, from health status to pathological conditions. This intense effort and the wealth of generated information are well summarized by the astonishing increase in the number of protein structures determined by X-ray crystallography, as deposited in the Protein Data Bank (Figure 1). Whereas, most of the structures provide only a static view of proteins, time-resolved crystallographic methods (Moffat, 1989; Hajdu, 1993; Hajdu et al., 2000; Schlichting and Chu, 2000) have further expanded the exploration of the protein conformational landscape. Along this effort, the very recent achievements using femtoseconds pulses from X-ray free electron lasers have allowed to detect early molecular events triggered by light on photosystem II (Kupitz et al., 2014) and photoactive yellow protein (Tenboer et al., 2014), opening new avenues to the understanding of protein dynamic and function.

Protein structures are a key information for a variety of research fields that exploit structural data to pursue very distinct goals, such as the elucidation of protein-protein interaction, the determination of enzyme catalytic mechanisms and allosteric regulations, the development of drugs via structure-based and computer-based methods, the comprehension of protein flexibility via molecular dynamics simulations, and the prediction of protein structures using homology modeling. Therefore, the correspondence between the protein structure determined in the crystal and the structure in solution is critical. There are at least three distinct issues that may affect the determination and interpretation of a crystal structure. First, a crystal structure is a model generated through a series of experimental and computational steps including the fitting of the electron density to the amino acid sequence, ligand and water molecules, and the energy minimization to remove steric clashes. In the model, the position of each atom is associated with a B factor that depends on atom mobility, and the protonation state of ionizable groups cannot be determined, except in cases where the resolution is better than about 1 Å. Second, there are many possible crystal “artifacts,” including effects due to the composition of the crystallization medium, lattice forces constraints, and X-ray radiation damage. Third, a native protein is an ensemble of many different conformations, generating the so called energy landscape (Frauenfelder et al., 1991; Bryngelson et al., 1995; Carlson, 2002; Boehr et al., 2009; Nussinov and Tsai, 2014). It is well known that the most stable and the most populated protein conformation is not necessarily the species that plays the most significant functional role. Furthermore, the protein conformation that is less soluble is likely to be selected in the crystallization process.

These considerations pose several questions:

- Which conformation is selected by crystallization and how active is it?
- How different is the crystallized conformation with respect to the conformation active in solution?
- How can the activity of a protein in a crystal be assessed in a quantitative way and compared with its activity in solution?

An answer to these questions for most of the protein structures deposited in the PDB is scarce. With more than 100,000 solved structures, functional/spectroscopic/dynamic measurements have been carried out for less than 100 proteins in the crystalline state. In turn, this implies that many protein function and regulation mechanisms are based on weak grounds and many studies aimed at developing drugs are exploiting a shaky target conformation.

## Assessing protein function in the crystalline state by spectroscopic methods

Functional properties in the crystalline state have been investigated over the years predominantly exploiting two distinct approaches: (i) measurements of enzyme activity on microcrystalline suspensions, and (ii) spectroscopic studies on single crystals. The former approach involves activity assays under conditions in which the enzyme is in the crystalline state and the crystal size is such that the rate of the catalytic reaction is not limited by reagent diffusion to and from enzyme active sites. The suitable crystal size is calculated by a formula developed several years ago at the dawn of structural biology (Hoogenstraaten and Sluyterman, 1969). This approach, rarely exploited, was used to evaluate the activity of microcrystals of papain (Hoogenstraaten and Sluyterman, 1969) tryptophan synthase (Ahmed et al., 1987), and pyridoxal 5′-phosphate (PLP)-dependent enzymes encapsulated in micrometer-size wet nanoporous silica gels (Pioselli et al., 2004, 2005).

The second approach relies on the spectroscopic techniques that are able to measure the dynamic and functional properties of protein single crystals of the same quality and size as those used for the structural determination. These include UV-vis absorption microspectrophotometry, microspectrofluorimetry, microRaman, and Resonance Raman, IR and EPR. Spectral changes of either endogenous or exogenous chromophoric probes are monitored, reflecting protein molecular events. A few reviews have been previously published summarizing the basic principles required for carrying out spectroscopic measurements on single crystals (Hofrichter and Eaton, 1976; Mozzarelli and Rossi, 1996; Pearson et al., 2004; Bourgeois and Royant, 2005; De La Mora-Rey and Wilmot, 2007; Pearson and Owen, 2009; Carey et al., 2011; McGeehan et al., 2011; Ronda et al., 2011b; Sage et al., 2011; von Stetten et al., 2015). Here, we will predominantly focus on UV-vis absorption microspectrophotometry because it is the most widely used approach.

The development of protein crystal optical spectroscopy was primarily carried out by a few laboratories (Eaton and Hochstrasser, 1967; Rossi and Bernhard, 1970; Berni et al., 1977; Eichele et al., 1978; Metzler et al., 1988) in order to obtain structure-function correlation from measurements carried out in the same physical state. With the increase in the number of solved protein structures, crystallographers understood the relevance and power of coupling X-ray diffraction data with spectroscopic measurements. This led to the development of on-line and off-line microspectrophotometers and optical spectroscopy laboratories at synchrotron sites. The first on-line microspectrophotometer was developed by Hajdu (1993) and applied to investigate the release of phosphate triggered by photolysis of a caged compound, 3,5-dinitrophenyl phosphate, in crystals of glycogen phosphorylase *b* (Hadfield and Hajdu, 1994). The first on-line and off-line optical laboratory was set-up at ESRF by Bourgeois and coworkers (Bourgeois et al., 2002), and further implemented by Garman (McGeehan et al., 2009) and Royant (von Stetten et al., 2015). Nowadays, single crystal spectroscopic instrumentations are present at most synchrotrons with beamlines dedicated to protein crystallography (Pearson et al., 2004; Pearson and Owen, 2009; Pearson and Mozzarelli, 2011; von Stetten et al., 2015) (Table 1). The on-line microspectrophotometer geometry varies significantly from site to site depending on the geometry of the beamline and specific needs. Some of the issues and potentialities of an on-line microspectrophotometer for UV-vis absorbance, fluorescence and Raman measurements have been very recently summarized for the instrumentation available at ESRF (von Stetten et al., 2015). However, it should be pointed out that not all on-line instrumentations work with linearly polarized light, a strong requirement for obtaining absorbance intensity strictly proportional to crystal thickness, chromophore concentration and extinction coefficients, i.e., spectra that obey to the Beer-Lambert law (Hofrichter and Eaton, 1976). When unpolarized light is used, only qualitative information is derived from spectra that are generally of lower quality. In any event, such unpolarized spectra are useful for determining the occurrence of a reaction, for monitoring the time course of metastable intermediates accumulation and breakdown and the redox state of a protein. This information is crucial for the definition of freeze-flashing times in cryo-crystallographic experiments. Furthermore, single crystal spectroscopic measurements are valuable to assess whether X-ray radiation has caused any undesired effect on protein crystals (Leiros et al., 2006 and references therein). Damages that can be spectroscopically detected are photoreduction of metals, such as ferric to ferrous iron conversion, and disulfide breakage (see below), whereas decarboxylation can only be assessed with structural methods, including mass spectrometry.

**Table 1:** **Single crystal spectroscopy instrument at synchrotron centers**.

**Synchrotron center, location**	**Web site**	**Available equipment**	**References**
Swiss Light Source (SLS), Villigen, Switzerland–Beamline X10SA (PXII)	http://www.psi.ch/sls/pxii/spectrolab	UV–vis absorption, Raman and fluorescence multimode spectrometer; on-axis geometry	Beitlich et al., 2007; Owen et al., 2009
BioCARS, Chicago, IL, USA–Beamline 14-BM-C	https://biocars.uchicago.edu/page/biology-customized-macromolecular-crystallography	On-line 4DX systems microspectrophotometer	De La Mora-Rey and Wilmot, 2007; Pearson et al., 2007
ESRF, Grenoble, France (MX diffractometers)	http://www.esrf.eu/UsersAndScience/Experiments/MX/Cryobench/Equipment/Microspec	CryoBench microspectrophotometer for UV-vis absorption, fluorescence and Raman measurements	Royant et al., 2007; McGeehan et al., 2009; von Stetten et al., 2015
National Synchrotron Light Source (NSLS), Upton, NY, USA–Beamline X26C	http://beamlines.ps.bnl.gov/beamline.aspx?blid=X26C	On-line 4DX System for visible absorption and Raman measurements	Orville et al., 2011; Stoner-Ma et al., 2011
Synchrotron Radiation Source (SRS) at the Daresbury Laboratory, UK–beamline 10	Recently decommisioned	UV-vis absorption measurements	Ellis et al., 2008
Diamond Light Source Oxfordshire, UK–MX beamline I02	http://www.diamond.ac.uk/Beamlines/Mx/Equipment-on-Demand/Spectroscopy.html	On line and off-line UV-vis absorption measurements, at the final stages of commissioning	–
SPring-8, Hyôgo Prefecture, Japan–BL38B1 beamline	http://www.spring8.or.jp/en/	UV-vis absorption measurements	Shimizu et al., 2013

## Examples of protein structure-function correlation in the crystalline state

In recent years single crystal optical spectroscopy measurements have been carried on several proteins, as reported in Table 2. Here, we report some representative investigations of protein function in the crystalline state carried out in our laboratory and a few studies carried out in other laboratories.

**Table 2:** **Proteins investigated by single crystal optical microspectrophotometry since 2011**.

**Protein**	**References**
Baeyer-Villiger monooxygenase	Orru et al., 2011
Catalase	Purwar et al., 2011
Methionine gamma lyase	Ronda et al., 2011a
Bacterioferritin	Antonyuk and Hough, 2011
Green Cu nitrite reductase	Antonyuk and Hough, 2011
Myoglobin	Hersleth and Andersson, 2011
Catechol 1,2 dioxygenase	Micalella et al., 2011
3-Isopropylmalate dehydrogenase	Graczer et al., 2011
Green fluorescent protein	Royant and Noirclerc-Savoye, 2011
Metalloproteins	Owen et al., 2012
Copper amine oxidase	Johnson et al., 2013
Lysozyme	Sutton et al., 2013
HbTb	Merlino et al., 2013
HbTb	Ronda et al., 2013b
Hb II	Ronda et al., 2013a
Hb	Shibayama et al., 2014
Bacteriorhodopsin	Borshchevskiy et al., 2014
Cystalysin	Spyrakis et al., 2014

*For earlier studies, see Mozzarelli and Rossi (1996), Pearson et al. (2004), and Pearson and Mozzarelli (2011)*.

### Hemoglobins

**Figure 2:**
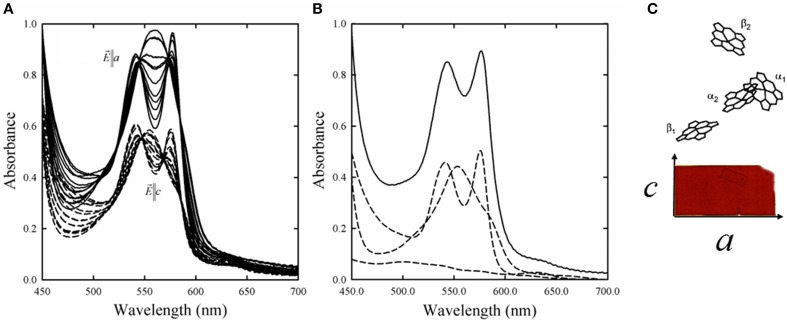
**(A)** Absorption spectra of Hb crystals collected as a function of oxygen pressure between 0 and 760 torr, with the electric vector of linearly polarized light parallel to *a* and *c* crystallographic axes of orthorhombic crystals grown from polyethylene glycol with dimensions of about 20 × 60 μm (Rivetti et al., 1993a). Spectra of deoxyHb crystals exhibit a peak at about 555 nm, whereas spectra of oxyHb exhibit peaks at about 541 and 577 nm. **(B)** Oxygen fractional saturation was calculated by fitting the observed spectrum (solid line), recorded at a defined oxygen pressure, to a linear combination (dotted line) of the reference spectra, deoxyHb, oxyHb, metHb, and a baseline (dashed lines) (Rivetti et al., 1993a). **(C)** Heme projection along the *a* and c crystal axis that leads to a higher absorbance intensity for spectra recorded along the *a* axis.

**Figure 3:**
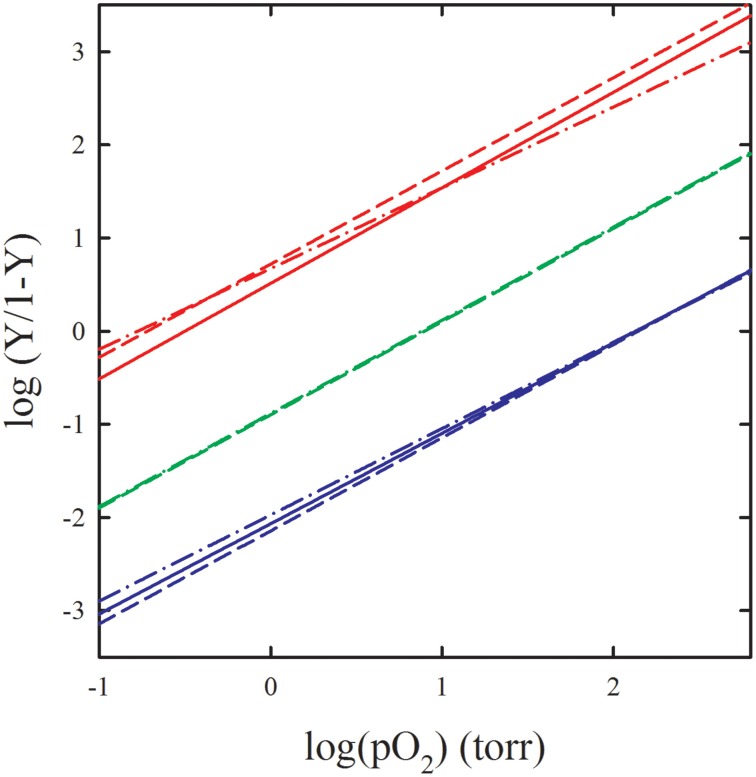
**Comparison of oxygen binding curves of HbA in the crystal, in gel and in solution**. Oxygen binding to: R state Hb C crystals (red continuous line) (Shibayama et al., 2011), R state Hb gels (red dot-dash line) (Shibayama and Saigo, 1995), R state Hb in solution (red dash-dash line) (Yonetani et al., 2002), T state Hb crystals (blue continuous line) (Mozzarelli et al., 1997), T state gels in the presence of allosteric effectors (blue dot-dash line) (Viappiani et al., 2004), Hb in solution in the presence of allosteric effectors (blue dash-dash line) (Yonetani et al., 2002), T state Hb gels in the absence of allosteric effectors (green dot-dash line) (Bruno et al., 2001a), T state Hb in solution in the absence of allosteric effectors (green dash-dash line) (Poyart et al., 1978).

The Monod, Wyman and Changeux (MWC) allosteric model was developed to explain the sigmoidal binding curves that characterize the interaction between ligands and some oligomeric proteins (Monod et al., 1965). Along the years, several studies challenged the validity of the MWC model applied to Hb (Eaton et al., 1999, 2007; Peracchi and Mozzarelli, 2011), either proposing its extension or completely discarding it. Key assumptions of the MWC model are that (i) only two quaternary states, T and R, exist and are endowed with different functional properties, and (ii) within each quaternary state, ligand binding is fully non-cooperative. In order to verify the validity of the MWC model oxygen binding curves were determined for Hb crystals constrained in the T state by lattice forces (Mozzarelli et al., 1991, 1997; Rivetti et al., 1993a; Bettati et al., 2009). Since Hb structures in the absence and presence of oxygen were obtained from crystals grown from polyethylene glycol of the same size and quality (Liddington et al., 1992; Paoli et al., 1996) as those used for the functional measurements, a straightforward structure-function relationship could be derived. Absorption spectra were recorded as a function of oxygen pressure by a single crystal microspectrophotometer (Figure 2A) using light linearly polarized along the *a* and *c* crystal axes of orthorhombic plates (Figure 2B) (Mozzarelli et al., 1991; Rivetti et al., 1993a). Fractional saturation was determined by fitting the observed spectra to a linear combination of pure deoxy-, oxy-, and metHb plus a baseline (Figure 2C) (Ronda et al., 2008). It was found that oxygen binding is non-cooperative, with a p50, i.e., the oxygen pressure at half-saturation, of 130–150 torr, at 15°C (Table 3). This affinity is the same as that for the first oxygen molecule that binds to Hb, determined in solution in the presence of strong allosteric effectors (Marden et al., 1990; Bruno et al., 2007). To evaluate the role of the salt bridges and residues at the α_1_β_2_ interface in controlling oxygen affinity, binding curves were determined for desArgHb (Kavanaugh et al., 1995), desHisHb (Bettati et al., 1997), Cowtown Hb (His β146Leu) crystals (Bettati et al., 1998), and for Hb Rothschild (Trp β37Arg) (Rivetti et al., 1993b), Tyr β35Phe, Tyr β35Ala (Kavanaugh et al., 2001), Asn β108Gly, Asn β102Ala, Tyr β35Ala, Trp β37Glu, and Tyr α42Ala (Noble et al., 2001) (Table 3). These measurements confirmed that oxygen binding to T state Hb is non-cooperative, a low affinity conformation is stabilized in the crystal, and His β146 plays a limited role in controlling oxygen affinity and a key role in the quaternary transition, as also recently proposed based on computational analyses (Fischer et al., 2011). Remarkably, the effect of mutations on the oxygen affinity detected for mutant Hb crystals was the same as that observed in solution for the binding of the first oxygen. Furthermore, a good correlation was observed between the p50 of these Hb mutants determined in the crystal and the rate of reaction of the first CO molecule with Hb in solution (Noble et al., 2002). Overall, the functional properties detected in T state Hb crystals were the same as in solution in the presence of strong allosteric effectors. The robustness of functional data obtained in the crystals was further evaluated by encapsulation of Hb in wet, nanoporous silica gels either in the T or R quaternary state, in the absence and presence of allosteric effectors (Shibayama and Saigo, 1995; Bettati and Mozzarelli, 1997; Abbruzzetti et al., 2001; Bruno et al., 2001a; Ronda et al., 2006). Protein encapsulation in silica gels is a powerful strategy to stabilize tertiary/quaternary states (Bruno et al., 2011). Crystal and gel Hb oxygen binding curves in the absence and presence of allosteric effectors fully agree (Figure 3). These equilibrium experiments triggered an extensive series of laser flash photolysis experiments of CO rebinding to Hb gels in the T and R state (Abbruzzetti et al., 2001; Viappiani et al., 2004, 2014) that support the Tertiary Two-State (TTS) model of Eaton and coworkers. This model extends the MWC taking into account preexisting tertiary equilibria partially uncoupled from quaternary states (Henry et al., 2002; Eaton et al., 2007). The TTS model has been further supported by resonance Raman spectroscopic studies on Hb gels monitoring the quaternary transition (Jones et al., 2012, 2014). It is interesting to note that investigations on Hb fixed in a defined quaternary state either by crystallization or encapsulation have allowed to unveil conformational properties that escape to notice in solution because of the confounding overlapping of binding and tertiary and quaternary relaxations.

**Table 3:** **Oxygen binding parameters for hemoglobin crystals**.

	**Conditions**	**p50^a^ (Torr)**	**Hill n^a^**	**References**
HbA	no allosteric effectors	136/133	1.00/1.01	Mozzarelli et al., 1997
HbA	+IHP	139/132	0.94/0.95	Mozzarelli et al., 1997
HbA	+BZF	138/127	0.94/0.97	Mozzarelli et al., 1997
des(αArg141)Hb		12.7/9.6	0.97/0.99	Kavanaugh et al., 1995
des(βHis146)Hb		81/76	0.98/1.01	Bettati et al., 1997
βTyr35Phe		157/148	0.88/0.91	Kavanaugh et al., 2001
βTry35Ala		79/80	1.16/1.15	Kavanaugh et al., 2001
βTrp37Arg (HbRothschild)		22/16	0.80/0.88	Rivetti et al., 1993b
βTrp37Glu		2.6	ND	Noble et al., 2001
βAsn102Ala		112	0.94	Noble et al., 2001
βAsn108Leu		145	ND	Noble et al., 2002
βAsn108Gly		80	1.15	Noble et al., 2001
βTyr145Ala		28	ND	Noble et al., 2002
βHis146Leu (HbCowtown)		44/45	0.99/0.98	Bettati et al., 1998
αTyr42Ala		33	1.06	Noble et al., 2001
α(Fe^2+^)_2_β(Ni^2+^)_2_		95/87	0.96/0.90	Bruno et al., 2000
α(Ni^2+^)_2_β(Fe^2+^)_2_		123/102	0.90/0.90	Bettati et al., 1996
α(Fe^2+^)_2_β(Zn^2+^)_2_		81/81	1.08/1.10	Samuni et al., 2003
α(Zn^2+^)_2_β(Fe^2+^)_2_		155/152	1.13/1.08	Samuni et al., 2003

a *p50 and Hill n were calculated from oxygen binding curves measured with light linearly polarized along two perpendicular crystal optical axes. ND, not defined. Measurements were carried out at 15°C*.

A remarkable investigation was recently carried out by determining the structure and oxygen binding affinity for nine equilibrium conformers of partially and fully ligated human β–β cross-linked [α(Fe^2+^-CO)β(Ni^2+^)][α(Ni^2+^)β-(Fe^2+^-CO)] Hb in the presence and absence of phosphate on three isomorphous crystals, covering the conformations of Hb from T to R2 (Shibayama et al., 2014). A previously unidentified intermediate conformer, between T and R, was also identified exhibiting an intermediate oxygen affinity. Whether these findings are revealing novel functional and conformational features of Hb remains to be established, given the conformational constraints of the cross-linking on the metal-hybrid Hb derivatives.

**Figure 4:**
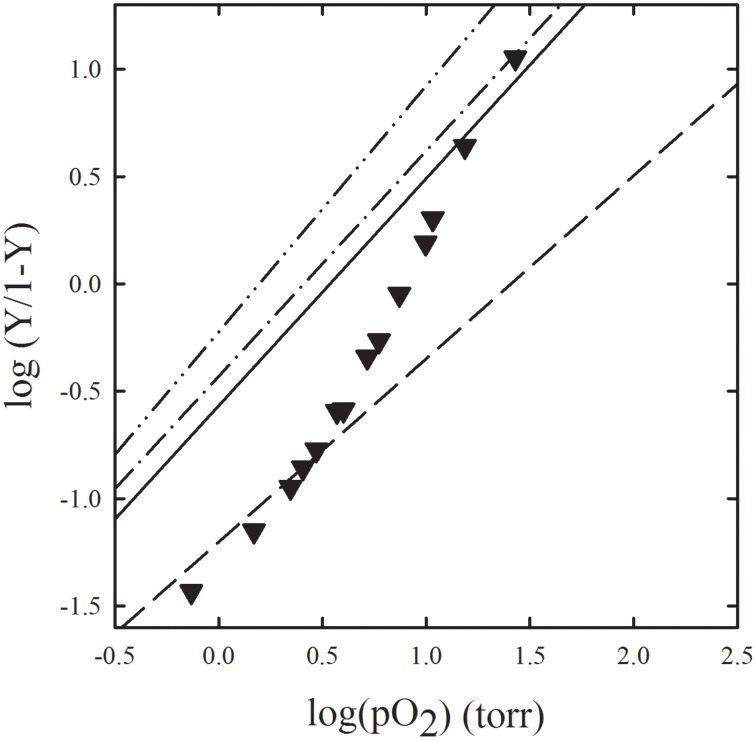
**Comparison of oxygen binding curves of HbII from *Scapharca inaequivalvis* in the crystal, in gel, and in solution**. Oxygen binding to: HbII crystals, grown in 2.2 M phosphate, measured with light linearly polarized along two perpendicular directions (dash-dot-dot line, and dash-dot), R state HbII gels (dash-dash line), T state HbII gels (continuous line), HbII in solution (closed inverted triangles) (Ronda et al., 2013a).

Microspectrophotometric studies on single crystals of fish Hbs, and of HbI and HbII from *Scapharca inaequivalvis*, represent good examples of functional and structural data that fully agree and led to straightforward structure-function correlation in the explanation of cooperative behavior (Ronda et al., 2013a). Oxygen binding curves were determined for crystals of the homodimeric HbI and found that HbI exhibits in the crystalline state the same cooperativity observed in solution (Mozzarelli et al., 1996). This finding is fully in keeping with the proposal based on structural data that purely tertiary conformational changes are responsible of the cooperative behavior (Chiancone et al., 1990). Oxygen binding curves of tetrameric HbII in the crystal as well as in gels were found to be apparently non-cooperative (Ronda et al., 2013a) (Figure 4). However, when the significant functional inequivalence of A and B chains was taken into account, both crystal and gel encapsulated HbII oxygen binding data were consistent with a tertiary contribution to cooperativity, quantitatively similar to that measured for HbI, as proposed on the basis of X-ray diffraction data. Furthermore, results indicate that to fully express the cooperative ligand binding, HbII also requires quaternary transitions hampered by crystal lattice and gel encapsulation.

In order to gain insight on the Root effect, a marked dependence of oxygen affinity and cooperativity on proton concentration observed in fish Hbs, structural and functional studies were carried out on crystals of deoxyHb from the Antarctic fish *Trematomus bernacchii* (HbTb) at pH 6.2 and pH 8.4 (Ronda et al., 2013b). Whereas, at low pH ligation causes negligible structural changes, an observation that correlates with low affinity and absence of cooperativity in oxygen binding, at high pH ligation causes significant tertiary changes within the T state. Oxygen binding curves of T-state HbTb crystals were consistent with the structural evidence. These findings indicate that, differently from mammalian Hbs, in HbTb a significant degree of cooperativity in oxygen binding is associated to tertiary conformational changes. The same HbTb crystals were investigated by Raman spectroscopy as a function of radiation dose to understand the stability of the nitrosylated derivatives (Merlino et al., 2013). It was found that radiation-triggered NO photodissociation causes a conformational transition at the beta chain forming a pentacoordinate species.

Overall, spectroscopic studies of Hbs crystals have allowed to unequivocally associate structural changes, crystallographically detected, with molecular events triggered by ligand binding and eventually linked to cooperativity, and to discriminate between function-related and function-unrelated structural changes.

### Enzymes

Single crystal microspectrophotometry has been applied to characterize the ligand binding and catalytic competence of many enzymes. In some cases, chromophoric substrate analogs have been used, whereas, in most of the cases, the signals generated by cofactors bound to the active site have been monitored as suitable reporter of molecular events associated with enzyme function.

Representative case-studies are the pyridoxal 5′-phosphate (PLP)-dependent enzymes cystalysin (Spyrakis et al., 2014) and methionine gamma lyase (Ronda et al., 2011a), and the iron-containing catechol 1,2 dioxygenase (Micalella et al., 2011).

PLP is the coenzyme of enzymes involved in the metabolism of amino acids, amines and ketoacids (Mozzarelli and Bettati, 2006). PLP spectral properties depend on enzyme-bound substrate, substrate analogs, inhibitors and catalytic intermediates, thus providing a signal for determining binding constants and rate of reactions. Several PLP-dependent enzymes have been investigated in the crystalline state, including aspartate aminotransferase (Eichele et al., 1978; Mozzarelli et al., 1979; Metzler et al., 1988), serine hydroxymethyltransferase (Schirch et al., 1981), tryptophan synthase (Mozzarelli et al., 1989), cystathionine beta-lyase (Bruno et al., 2001b), *O*-acetylserine sulfhydrylase (Mozzarelli et al., 1998), DOPA decarboxylase (Peracchi et al., 1994) and GABA aminotransferase (Storici et al., 2004).

**Figure 5:**
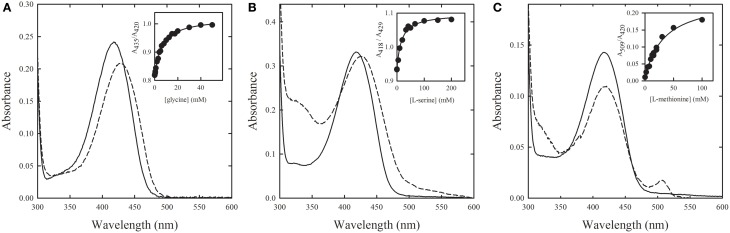
**Binding of substrate analogs to PLP-dependent cystalysin crystals**. Absorption spectra were recorded in the absence (solid line) and presence (dashed line) of saturating concentrations of **(A)** glycine, **(B)** L-serine, and **(C)** L-methionine. Insets: fitting of titration data points to binding isotherms with K_d_ of **(A)** 6.3 ± 0.3 mM, **(B)** 16 ± 2 mM, and **(C)** 33 ± 5 mM (Spyrakis et al., 2014).

Cystalysin catalyzes the breakdown of cysteine in pyruvate, ammonia and sulfide. The enzyme is considered a virulence factor in adult periodontitis since sulfide contributes to hemolysis sustaining pathogen survival and proliferation in the gingival crevice. For this reason, cystalysin is a promising target for antibiotic agents (Amadasi et al., 2007). The three-dimensional structure of the enzyme has been determined in the absence and presence of an aspecific covalent inhibitor, AVG (Krupka et al., 2000). In order to exploit these structures for a virtual screening campaign aimed at identifying potential active site inhibitors, cystalysin structure was first validated by determining spectral properties and ligand binding using single-crystal absorption microspectrophotometry (Spyrakis et al., 2014). Overall, the enzyme in the crystal and in solution exhibits the same absorption spectra for the catalytic intermediates, similar pK_a_ values for the residue controlling the formation of ketoenamine species, and close dissociation constants for glycine, serine and methionine (Figure 5). Upon this validation step, the cystalysin structure was used in a virtual screening, carried out using FLAP (Baroni et al., 2007). A list of compounds predicted to act as reversible, non-covalent active site inhibitors was obtained. Compounds were docked in cystalysin active site using GOLD (Jones et al., 1995), their interaction energy was scored using HINT (Kellogg et al., 2001; Spyrakis et al., 2004, 2007a,b; Amadasi et al., 2006; Marabotti et al., 2008; Salsi et al., 2010), and compound-enzyme complexes were visually inspected. The top 17 compounds were selected and assayed in solution, identifying two inhibitors with K_i_ of 25 and 37 μM (Spyrakis et al., 2014).

**Figure 6:**
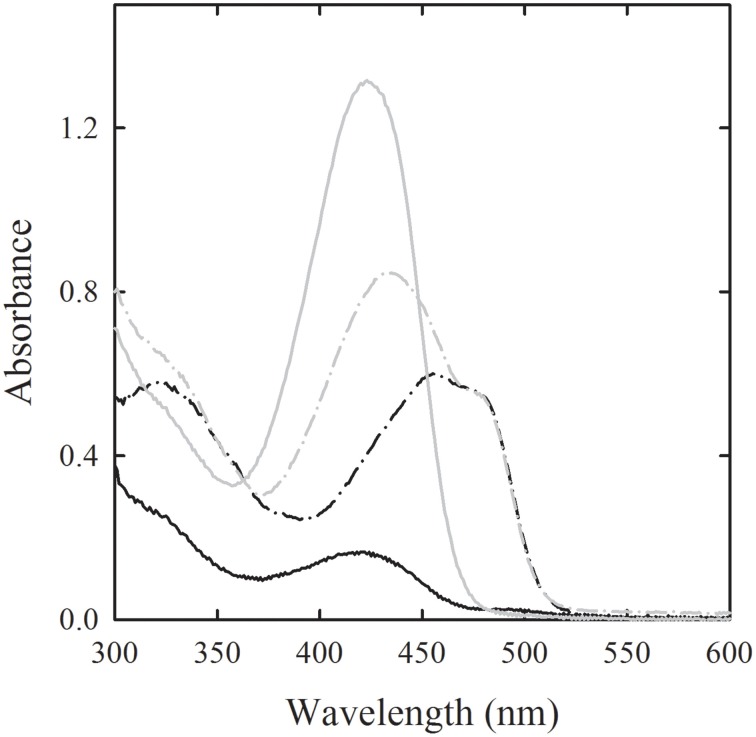
**Binding of methionine to PLP-dependent methionine gamma lyase crystals**. Polarized absorption spectra were recorded along two perpendicular directions (light and dark gray) for the enzyme in the absence (solid lines) and presence of 100 mM L-methionine (dash-dot line) (Ronda et al., 2011a).

A single crystal microspectrophotometric study (Ronda et al., 2011a) was carried out on methionine γ-lyase (MGL), an enzyme that catalyzes the γ-elimination reaction of L-methionine to produce α-ketobutyric acid, methanethiol and ammonia. Free and PEGylated MGL are potential biopharmaceutical drugs against cancer because cancer cells exhibit a strong dependence on methionine and delivered MGL is able to reduce the methionine level in the cell medium (Takakura et al., 2006). Furthermore, MGL can activate the pro-drug trifluoromethionine that is a recognized antibiotic agent (Coombs and Mottram, 2001). To be applied in cancer therapy, MGL needs to be genetically engineered in order to improve its catalytic efficiency and stability, whereas to be used as an effective pro-drug activator for antibiotic therapy its active site needs to be fully mapped in order to design novel, more efficiently activated pro-drugs. Therefore, in both cases, the availability of the three-dimensional structure of wild type MGL is a fundamental requirement. In order to validate MGL structure, enzyme crystals were reacted with the substrate methionine (Figure 6), the substrate analog vinylglycine, and the competitive inhibitors glycine and cycloserine, monitoring the reaction with single crystal microspectrophotometry (Ronda et al., 2011a). The observed dissociation constants were found to be slightly higher than in solution, pointing to some changes in the conformational distribution in the crystal with respect to solution. This finding suggests that this MGL structure should be considered with some caution and further activities should be carried out for the crystallization of fully active MGL. Furthermore, the three-dimensional structure of the native enzyme, determined from crystals grown in PEG, in the presence of ammonium sulfate, revealed the absence of the aldimine bond between the active site Lys210 and PLP, whereas absorption spectra collected for the same enzyme crystals were consistent with the presence of the aldimine bond. Different hypothesis were proposed and discussed in the light of spectral and structural data.

Catechol 1,2 dioxygenase is a Fe (III)-dependent enzyme that catalyzes the oxygenation of catechol and substituted rings. The Fe (III) is coordinated in a trigonal-bipyramidal geometry by two histidine and two tyrosine residues and a hydroxyl ion in the equatorial plane. When catechol binds at the active site, the axial tyrosine and the hydroxyl ligand are displaced, allowing a direct coordination of the diol to the Fe(III). The enzyme exhibits a broad band centered at around 440 nm, indicative of the ligand-to-Fe(III) charge transfer transition, typical of tyrosinate coordination to the Fe ion. When the enzyme binds cathecol in an improductive fashion (i.e., under anaerobic conditions), a decrease in intensity of the 440 nm band is observed. In view of determining the structure of isolated intermediates, polarized absorption spectra were collected on single crystals of catechol 1,2 dioxygenase from *Acinetobacter radioresistens* (Ar-1,2-CTD) (Micalella et al., 2011). The maintenance of the metal coordination in the crystalline state was thus confirmed. As in solution, the unproductive binding of catechol was observed under anaerobic conditions. Spectra were also correlated with the three dimensional structures of the wild type and two mutants exhibiting higher specificity for chloro cathecols and designed as the basis for bioreactors to be used in bioremediation (Micalella et al., 2014). Overall, the consistency of the spectroscopic properties of Ar-1,2-CTD in solution and in the crystalline state indicated that Fe(III) coordination and ligand binding observed through X-ray crystallography reflected those of the enzyme in solution.

### Green fluorescent proteins

The green fluorescent protein (GFP) from *Aequorea victoria* is the prototype of a large family of fluorescent proteins (FPs) from marine organisms, displaying a genetically encoded bright fluorescence in several regions of the visible spectrum (Cubitt et al., 1995; Pakhomov and Martynov, 2008). Moreover, FPs have been extensively engineered to produce hundreds of variants characterized by distinct fluorescent bands, emitting from yellow to cyan, often endowed with pH and redox-sensitive properties (Zhang et al., 2002; Remington, 2011). The endogenous chromophore is formed upon an internal, autocatalytic, post-translational modification (cyclization and oxidation) involving three conserved amino acids. A key determinant of FPs spectral variability is the nature and state of ionization of residues surrounding the endogenous chromophore (Tsien, 1998).

Despite the wealth of structural information (from X-ray diffraction and, in part, NMR studies) and functional data in solution, only a few absorption, fluorescence, and Raman spectroscopy studies have been carried out on single crystals of FPs (Bettati et al., 2011). Pioneer studies by Ward and coworkers exploited steady-state and time-resolved fluorescence to ascertain that crystallization does not induce significant structural distortions (Perozzo et al., 1988). Several years later, two different groups exploited fluorescence polarization (Inoue et al., 2002) and polarized light absorption microspectrophotometry (Rosell and Boxer, 2003; Royant and Noirclerc-Savoye, 2011) to extract information on the geometric relationships between chromophore transition dipole moment directions, the crystal axes and protein coordinates. This kind of information is of high potential interest for spectroscopic applications relying on geometric factors and their function-associated variations, like, e.g., Forster Resonance Energy Transfer (FRET). A few other groups exploited crystal spectroscopy to validate the correlation between crystal structures and spectroscopic properties in solution (Battistutta et al., 2000; Malo et al., 2007). However, the most impressive effort to couple single crystal spectroscopy to crystallographic studies of FPs has been carried out in recent years by Bourgeois and coworkers (Royant et al., 2007; Adam et al., 2008, 2009; Lelimousin et al., 2009; Violot et al., 2009). The dedicated absorption and fluorescence spectrometers assembled at the European Synchrotron Radiation Facility of Grenoble, France, is equipped with a Time-Correlated Single-Photon Counting system to measure fluorescence lifetimes in the crystalline or solution state, at room and cryo temperatures, and a Raman probe that can be used off-line of the microspectrophotometer or on-line of diffractometers (McGeehan et al., 2011). This apparatus has been exploited to investigate the structural basis of X-ray or visible light-induced photobleaching of IrisFP, a mutant of EosFP capable of different phototransformation properties as irreversible green-to-red photoconversion and reversible photoswitching between fluorescent and dark states (Adam et al., 2008, 2009; Duan et al., 2013, 2014).

With the goal of a better understanding of the interplay between dynamics of the protein matrix and GFP photochemistry and photophysics, with special regard to acid-base properties, our group is currently investigating the pH dependence of the absorption spectra on crystals of GFPmut2 (Ser65Ala, Val68Leu, Ser72Ala GFP), grown in alkaline or acidic conditions. Both crystals show reversible, pH-dependent spectral changes, with pK_a_ values and shapes of the transition curve that differ from each others, and from the pH-dependence determined in solution. These results are driving ongoing X-ray diffraction studies aiming at determining the structural basis of the observed behavior and the role of key residues known to affect GFP structural dynamics and chromophore protonation state. These activities, once again, highlight the necessity to correlate structural and functional information collected in the same physical state.

## Detection of protein crystal X-ray radiation damage by spectroscopic methods

Many investigations have reported that the intense X-ray beams cause damages to proteins, casting doubts on the quality of the solved protein structures and posing limitations to high-resolution structural determination. Therefore, studies have analyzed which are the main factors leading to protein radiation damages (Garman and Weik, 2013).

The issue of radiation damage, well known since the inception of protein crystallography, was limited for years by acquiring data at cryo-temperature. However, both (i) the emerging of third generation synchrotron sources, able to deliver much higher radiation doses, and (ii) an increased interest in room temperature X-ray data collection as a way to observe biologically relevant conformations, caused an increase in the extent of radiation damages. This, in turn, led to a renewed interest in the factors influencing them. Specifically, it is widely recognized that temperature has a relevant effect on determining the extent of X-ray-induced damages, and the effective damage is markedly dependent on the total number of deposited photons (Davis et al., 2013).

Many complementary techniques, such as X-ray-excited optical luminescence of protein crystals (Owen et al., 2012), electron paramagnetic resonance (EPR) (Utschig et al., 2008), UV–visible absorption and X-ray absorption spectroscopy (XAFS) (Antonyuk and Hough, 2011), have been coupled to crystallography to better understand the processes involved in radiation damage with the aim of providing practical recommendation for the optimization of data collection conditions (Pearson et al., 2007; Hersleth and Andersson, 2011). A possible strategy for mitigating the radiation damage during protein crystallography data acquisition is the addition of radical scavengers at the crystallization stage or by soaking of crystals in radicals scavenger solution prior to data collection. This approach provided inconsistent results, mainly for discrepancies in metrics for evaluating damages and for the variability in the reactions of the crystallization medium with radical scavengers, as demonstrated by on-line microspectrophotometry (Allan et al., 2013). Here, we report a few representative cases of investigations exploiting protein crystal spectroscopy and X-ray crystallography to assess radiation damages.

By combining EPR, on-line UV-visible absorption microspectrophotometry and X-ray crystallography measurements, the effect of X-ray radiation on lysozyme crystals was carefully investigated. EPR showed a disulfide bond radicalization at ~0.2 MGy dose, lower than the 0.5–0.8 MGy saturating dose observed through UV-visible miscrospectrophotometry for disulfide bond damaging. This study demonstrated that disulfide bonds are reduced under a radiation dose regime that is usually applied for data collection (Sutton et al., 2013). In a similar study, the dose-dependent radiation damage was evaluated for bacteriorhodopsin (Borshchevskiy et al., 2014).

Metal centers in metalloproteins are particularly sensitive to radiation damage. Photoreduction is critical when redox proteins are investigated because proteins are in the oxidized state at the beginning and become reduced at the end of data collection. This, in turn, might cause conformational changes and uncertainty in the structure-redox state relationship of the protein and derived mechanisms. A very recent paper (Kekilli et al., 2014) reports a detailed investigation of hemoprotein crystals exposed to synchrotron radiation by using resonance Raman spectroscopy. Furthermore, bovine catalase crystals in the absence and presence of ammonia and nitric oxide were investigated by X-ray crystallography and on-line microspectrophotometry, revealing photoreduction of the central heme iron (Purwar et al., 2011). Photoreduction of redox-active protein cofactors has been also studied for Mn ions of oxygen-evolving complex of photosystems II (PSII) by using X-ray emission spectroscopy with wavelength-dispersive detection (Davis et al., 2013). Mn ions, contained in PSII active sites, are in the Mn (III) and Mn(IV) oxidation state and are known to undergo during X-ray irradiation to the photoreduction to Mn(II) and to the cleavage of Mn di-μ-oxo units (Yano et al., 2005). PSII radiation damage was studied determining a kinetic model at different experimental rates of dose deposition and excitation wavelength. It was observed that high dose deposition rate could be beneficial in terms of reducing radiation damages in sensitive samples, although high rate of dose deposition could generate other radicals or initiate other processes (Davis et al., 2013).

Structures of T6 bovine insulin complexed with Ni(^2+^) and Cu(^2+^) were solved using a synchrotron radiation, showing a deterioration of the coordination of water for Cu (II) site of the insulin derivative due to radiation damage. X-Ray Absorption spectra (XAS) and EPR spectroscopy were used to obtain information on the metal coordination and the metal redox state. It was observed that in the insulin copper derivative, during radiation-induced photoreduction, the coordination geometry changes toward lower coordination numbers. Different damages were studied as a function of the dose of radiation with different tehniques, i.e., photoreduction was monitored by XANES, while a diffractometer was used to follow structural changes around Cu atoms. The solid embedment of Cu insulin in a saccharose matrix partially suppressed the photoreduction at 100 K, and a further 30% suppression was obtained by cooling the samples to 20 K (Frankaer et al., 2014).

## Controversies and synergies between X-ray crystallography and single crystal UV-Vis microspectrophotometry

Here, we discuss representative cases of strong discrepancies between straightforward results from functional studies carried out in the crystal and structurally-derived functional interpretation, and representative cases were microspectrophotometric measurements were instrumental to the structural determination.

The first case is the structure of partially liganded human Hb where apparently only the α subunits showed bound oxygen. It was concluded that the Hb tetramer was functionally strongly asymmetric with oxygen loading and unloading only by α hemes (Brzozowski et al., 1984). Since polarized absorption spectra measured along the *a* and *c* crystal axes depend on the sum of the projections of the α and β hemes along these axes, and α and β hemes contribute differently along the crystal optical axes, it was possible to calculate separate oxygen binding curves and to determine that α subunits bind oxygen with an affinity about two fold higher than the β subunits (Mozzarelli et al., 1997). This finding is fully consistent with solution studies on mixed metal hybrid Hb, α(Fe^2+^)_2_β(Ni^2+^)_2_, α(Ni^2+^)_2_β(Fe^2+^)_2_, α(Fe^2+^)_2_β(Zn^2+^)_2_, αZn^2+^)_2_β(Fe^2+^)_2_ (Shibayama et al., 1993; Miyazaki et al., 1999), as well as on mixed metal hybrid Hb crystals (Bettati et al., 1996, 2009; Bruno et al., 2000). These findings cannot be reconciled with any structure-based hypothesis of a high difference in oxygen affinity between α and β subunits, and hint to crystallographic pitfalls.

The second case is based on the comparison between liganded and unliganded T state Hb in the presence of allosteric effectors. It was concluded that oxygen binding is associated with intersubunit communication/conformation stabilization, responsible for cooperativity within the T state (Paoli et al., 1997). However, oxygen binding curves to Hb in the crystals were systematically characterized by a Hill coefficient close to one (Table 3) (Bettati et al., 2009), indicating absence of cooperativity within the T state. This result was confirmed on all Hb crystals that were analyzed, including metal hydrids and mutants (Table 3). The relevant and general conclusion from these two cases is that not all structural changes crystallographically detected are associated with functional roles. Therefore, some caution should be used in the “straightforward” exploitation of structural data for proposing protein mechanisms.

The first example of a very tight complementarity between microspectrophotometric measurements and X-ray crystallography is represented by the NAD-dependent glycolytic enzyme glyceraldehyde 3-phosphate dehydrogenase (GAPDH). This enzyme was one of the first to be structurally characterized, by Rossmann and co-workers (Moras et al., 1975), and one of the first to be investigated by microspectrophotometry (Berni et al., 1977; Mozzarelli et al., 1982). GAPDH catalyzes the oxidation of glyceraldehydes-3-phosphate to 1,3-bisphosphoglycerate in the presence of NAD^+^ and phosphate. In spite of intense investigations carried out over several decades, the structural determination of the catalytic acyl-enzyme intermediate remained elusive. By microspectrophotometric studies monitoring at 340 nm the formation of NADH on single crystals of GAPDH from *Bacillus stearothermophilus* the experimental conditions for the accumulation of the metastable acyl-enzyme intermediate were determined (Moniot et al., 2008). On the basis of these results, GAPDH crystals were soaked with the substrate at the required substrate concentration for a defined time before flash-freezing. The structural determination of the thioacyl-enzyme allowed to propose a novel catalytic mechanism where the C3-phosphate group of the substrate changes its conformation concomitantly or after the redox step.

The second representative case of the complementary role of single crystal spectroscopy and X-ray crystallography is the investigation of the flavin-dependent enzyme phenylacetone monooxygenase (PAMO) from *Thermobifida fusca* (Orru et al., 2011). PAMO catalyzes the enantioselective Baeyer-Villiger oxidation and sulfoxidation of phenylacetone as well as other substrates. Single crystal UV-vis spectra of the enzyme, collected prior and after reduction and cooling at 100 K, and after melting and exposure to air, allowed to probe the redox state of flavin and the catalytic competence of the enzyme. Furthermore, spectra of PAMO crystals, recorded on-line with X-ray data collection, evidenced the progressive reduction of the flavin as a function of X-ray exposure, thus defining the time window for the structural determination.

Another interesting example of the combination of X-ray crystallography and absorption, fluorescence and Raman single crystal spectroscopy is the investigation of the molecular mechanism of the unusually large stokes shift of mKeima, a monomeric red fluorescent protein (Violot et al., 2009). Raman spectra on mKeima in the crystal state allowed to rationalize the peculiar pH dependence of absorption bands, supporting a “reverse protonation” effect consistent with crystallographic data collected at different pH values. Opposite to the normal behavior of GFP-like proteins, cis and trans chromophore conformations dominate at acidic and alkaline pH, respectively.

## Conclusions

Single crystal spectroscopies provide key functional information on proteins in the crystalline lattice, allowing to assess whether lattice forces, conformational selection and X-ray exposure have caused the emergence of “artifactual” structures. These investigations are of paramount relevance given the variety of scientific fields that exploit the structural data for the understanding of protein function or for the development of novel drugs. The increasing use of on-line and off-line crystal spectrometers at synchrotron sources clearly indicates that only via a tight link between X-ray crystallography and spectroscopy meaningful and robust protein structure and function correlation can be proposed. Within this frame, single crystal optical spectroscopy has been demonstrated to be by far the most exploited and the most powerful technique.

## Author contributions

LR, SB, SB, and PS contributed to the writing and figure preparation, AM conceived the work and contributed to the writing.

### Conflict of interest statement

The authors declare that the research was conducted in the absence of any commercial or financial relationships that could be construed as a potential conflict of interest.

## Abbreviations

PDB: protein data bank
FP: fluorescent protein
Hb: hemoglobin
PLP: pyridoxal 5′-phosphate
MGL: methionine γ–lyase.
